# Strategic sequencing of bladder-sparing therapies in the management of BCG-unresponsive non-muscle invasive bladder cancer

**DOI:** 10.1080/07853890.2025.2612387

**Published:** 2026-01-08

**Authors:** Mohamad Abou Chakra, Joost L. Boormans, Dickon Hayne, Michael A. O’Donnell

**Affiliations:** ^a^ Department of Urology, University of Iowa Health Care, Iowa, USA; ^b^ Department of Urology, Erasmus University Medical Center Cancer Institute, Rotterdam, The Netherlands; ^c^ UWA Medical School, University of Western Australia, Perth, Australia; ^d^ Department of Urology, Fiona Stanley Hospital, Murdoch, Western Australia, Australia

Intravesical Bacillus Calmette-Guérin (BCG) therapy remains the cornerstone of treatment for high-risk (HR) non-muscle-invasive bladder cancer (NMIBC), with maintenance regimens typically extending over 1 to 3 years to maximize therapeutic efficacy [1,2]. Nevertheless, approximately 40% of treated patients ultimately develop unresponsiveness to BCG [3]. Importantly, the U.S. Food and Drug Administration (FDA) defines BCG-unresponsive NMIBC as the presence of at least one of the following criteria: persistent high-grade (HG) T1 disease detected at the first evaluation, typically around 3 months after initiating treatment; recurrence of carcinoma *in situ* (CIS) within 12 months; or recurrence of HG Ta or T1 disease within 6 months, all occurring after adequate BCG therapy. Adequate BCG therapy is defined as receiving at least 5 out of 6 doses during the induction phase, along with either a minimum of 2 out of 3 doses of maintenance therapy or at least 2 out of 6 doses of re-induction instillations [4]. In such cases, clinical guidelines, including those issued by the European Association of Urology (EAU), American Urological Association (AUA), National Comprehensive Cancer Network (NCCN), and the International Bladder Cancer Group (IBCG), uniformly recommend radical cystectomy (RC) as the definitive intervention [1,2,4,5]. Although RC is effective, it is a major surgical procedure associated with significant risks. The overall complication rate within 90 days reaches 58.5%, ranging from 36.1% to 80.5%, and the 90-day mortality rate is 4.7% [6]. Due to these risks and the impact of radical surgery on quality of life [7], many patients either decline RC or are medically unfit for surgery. As a result, bladder-sparing therapies (BSTs) are often considered the only viable alternative for managing BCG-unresponsive disease.

An important consideration in the management of HR NMIBC has been the global shortage of Bacillus Calmette-Guérin (BCG), which began in 2012 and prompted the adoption of alternative intravesical therapies to mitigate treatment disruptions [8]. Among the most widely utilized BSTs are intravesical chemotherapeutic agents, including single-agent regimens such as gemcitabine (Gem) and mitomycin C (MMC), as well as sequential doublet therapies such as gemcitabine/docetaxel (Gem/Doce) and gemcitabine/mitomycin C (Gem/MMC) [9]. Currently, 4second-line therapies have received FDA approval for the treatment of BCG-unresponsive NMIBC. These include intravesical valrubicin (approved in 1998), systemic pembrolizumab (Pembro, approved in 2020), intravesical nadofaragene firadenovec (Adstiladrin), a non-replicating adenoviral vector-based gene therapy approved in 2022, and intravesical nogapendekin alfa inbakicept-pmln (Anktiva), approved in 2024 [10].

Despite the growing number of therapeutic options for BCG-unresponsive disease, guideline recommendations regarding optimal agent selection remain variable. Some guidelines offer broad or non-specific guidance, while others provide more detailed recommendations based on tumor histology, such as papillary versus carcinoma *in situ* (CIS). The AUA recommends intravesical chemotherapy, such as Gem/Doce or Gem, as well as nadofaragene or systemic Pembro for BCG-unresponsive NMIBC, while specifically endorsing Pembro only for BCG-unresponsive CIS [1]. In contrast, the EAU suggests a broader range of options, including intravesical chemotherapy (e.g. Gem/Doce or MMC combined with microwave-induced hyperthermia or electromotive drug administration), nadofaragene, and systemic Pembro, but does not provide specific guidance on how to select among these therapies [2]. The NCCN offers more granular recommendations. For both CIS and papillary disease, it endorses intravesical chemotherapy (e.g. sequential Gem/Doce), nadofaragene (with a category 2a recommendation for CIS and 2b for papillary disease), and systemic Pembro, which shares the same categorization as nadofaragene [5]. The IBCG has published the most detailed guidance on BSTs, emphasizing the importance of accurate disease staging, standardized definitions of BCG-unresponsive disease, and shared decision-making that incorporates toxicity profiles, efficacy data, quality-of-life considerations, and treatment accessibility when selecting among BSTs. For CIS, the IBCG recommends intravesical Gem/Doce, nadofaragene, Anktiva, and systemic Pembro in patients who have exhausted other options. For papillary disease, the panel supports intravesical Gem/Doce, nadofaragene, Anktiva, hyperthermic MMC, single-agent chemotherapy (Gem, MMC), and systemic Pembro. However, it notes that nadofaragene, Anktiva, and Pembro are not formally approved for papillary disease, though they may be considered based on emerging clinical data. Importantly, the IBCG underscores the need for reassessment following each therapeutic failure and emphasizes that patients should be counseled on the continued role of RC as the standard of care, even when BSTs are pursued [4].

A growing number of phase II and III clinical trials investigating novel agents in the BCG-unresponsive setting have recently been completed. Among the most prominent are TAR-200, a Gem-releasing intravesical device, and cretostimogene (CG0070), an oncolytic immunotherapy evaluated either as monotherapy or in combination with systemic pembrolizumab [11–13]. To properly assess the efficacy of previously FDA-approved agents and emerging therapies in this setting, it is essential to refer to benchmarks established by the IBCG. These benchmarks recommend a clinically meaningful initial complete response rate (CR) (for carcinoma *in situ* (CIS)) or recurrence-free rate (RFS) (for papillary tumors) of at least 50% at 6 months, 30% at 12 months, and 25% at 18 months [14].

In the management of BCG-unresponsive disease with CIS, various novel therapeutic agents have demonstrated differing levels of efficacy in achieving CR (Table 1). Among these, the combination of intravesical cretostimogene and systemic Pembro (CORE-001 trial) yielded the highest CR rate at any time, reaching 83%, followed by TAR-200 (SunRISe-1) at 82% and cretostimogene monotherapy (BOND-003) at 75%. Anktiva (QUILT 3.032) achieved a CR rate of 71%, while nadofaragene (NTC02773849) reported a notably lower CR of 55%. Longitudinally, the combination of cretostimogene and Pembro maintained superior CR durability, with rates of 57% at 12 months and 51% at 24 months. In contrast, cretostimogene monotherapy showed a decline to 46% and 42% at 12 and 24 months, respectively. Other agents, such as Anktiva and TAR-200, demonstrated intermediate durability, with CR rates of 45% and 46% at 12 months, and 37% and 42% at 24 months, respectively. Nadofaragene showed sustained CR rates of 24% at 12 months and 19% at 24 months. Systemic Pembro monotherapy (KEYNOTE-057) exhibited the lowest sustained CR, with only 19% at 12 months and 16% at 24 months. These results suggest that cretostimogene (alone or in combination with systemic Pembro), TAR-200, and Anktiva are the most promising agents in terms of sustained CR at 12 months, meeting the IBCG benchmark for clinically meaningful efficacy. Variability in biopsy protocols across clinical trials may significantly affects the accuracy of reported CR rates. Trials such as CORE-001, NCT02773849, and QUILT 3.032 included mandatory biopsies at predefined intervals, 12, 12, and 3 months, respectively, enhancing histological confirmation of treatment response. In contrast, KEYNOTE-057 did not include scheduled biopsies, relying solely on cystoscopic and cytological assessments, which may overestimate CR due to the potential for undetected CIS. The SunRISe-1 trial conducted required biopsies only at weeks 24 and 48, while most retrospective studies evaluating Gem/Doce included biopsies at 3 months; all studies permitted biopsies when clinically indicated. These protocol-specific requirements make it difficult to directly compare results with other trials and may introduce bias. Additionally, urologists should be aware that while “CR at any time” has recently gained traction as a clinical endpoint, it may be misleading. This metric can capture transient responses that do not reflect durable disease control, particularly in patients with fluctuating CIS status, potentially skewing the interpretation of treatment efficacy.

**Table 1.** Comparative efficacy of new agents used to treat NMIBC including that that are FDA approved.

Trial	BOND-003	CORE-001	QUILT 3.032	NTC02773849	KEYNOTE 057	SunRISe-1	Retrospective cohorts Gemcitabine/Docetaxel
Intervention	CG0070 (Cretostimogene)	CG0070+ Pembrolizumab	N-803 + BCG (Anktiva)	Adstiladrin (Nadofaragene)	Pembrolizumab	TAR-200	Sequential Gemcitabine/Docetaxel
Mechanism of action	Dual-action oncolytic immunotherapy that selectively replicates in cancer cells to destroy them while stimulating cytokine and tumor antigen release	Oncolytic immunotherapy + checkpoint inhibitor	Immune amplifier by activating NK and T cells	Delivery of gene encoding human IFNα2b	T-cell-mediated antitumor immune responses (immune check point inhibitor)	Gemcitabine-eluting device (sustained local exposure to chemotherapy)	DNA/RNA synthesis inhibition (Gemcitabine) followed by microtubule stabilization (Docetaxel)
Route	Intravesical	Intravesical + Intravenous	Intravesical	Intravesical	Intravenous	Intravesical	Intravesical
Therapy Regimen	Induction: Weekly × 6; Maintenance: Weekly × 3 Q 3 months (Year 1), then Q 6 months (Year 2)	Cretostimogene Induction: (1 × 10¹² viral particles) Weekly × 6; Maintenance: Weekly × 3 Q 3 months (Year 1), then at 18 months Pembrolizumab 400 mg IV was administered Q 6 weeks for approximately 2 years or until disease persistence or recurrence	400 μg of Anktiva with 50 mg BCG, once weekly for 6 weeks, then once a week for 3 weeks at months 4, 7, 10, 13, and 19; additional maintenance courses may be given at months 25, 31, and 37	75 mL at 3 × 10¹¹ viral particles/mL; once Q 3 months for up to 12 months (may continue if no HG recurrence)	Pembrolizumab 200 mg IV Q 3 weeks, up to 24 months or until confirmed disease persistence, recurrence, or progression	Administered every 3 weeks for 6 months, then quarterly for 2 years or until confirmed disease persistence, recurrence, or progression.	Gemcitabine 1000 mg/50 mL for 60 min, followed by docetaxel 37.5 mg/50 mL for 60 min; induction weekly ×6, maintenance monthly up to 2 years
Stage	Phase III	Phase II	Phase II	Phase III	Phase II	Phase II	Real world-multiple retrospective cohorts
Sample Size	N = 112 (CIS cohort)	N = 35 (CIS cohort)	N = 84 (CIS cohort) N = 77 (papillary cohort)	N = 103 (CIS cohort) N = 48 (papillary cohort)	N = 96 (CIS cohort) N = 132 (papillary cohort)	N = 85 (CIS cohort) N = 52 (papillary cohort)	N = 286 (CIS cohort) N = 241 (papillary cohort)
CR any time	75% (CIS cohort)	83% (CIS cohort)	71% (CIS cohort)	55% (CIS cohort)	N/A	CR = 82% (CIS cohort)	N/A
Efficacy outcomes at 3 mo	68% (CIS cohort)	82% (CIS cohort)	CR = 55% (CIS cohort)	CR = 53% (CIS cohort) HG-RFS = 73% (papillary cohort)	CR = 41% (CIS cohort)	CR = 79% (CIS cohort)	N/A
Efficacy outcomes at 12 mo	46%* (CIS cohort)	57% (CIS cohort)	CR = 45% (CIS cohort) DFS = 55% (papillary cohort)	CR = 24% (CIS cohort) HG-RFS = 44% (Papillary cohort)	CR = 19%* (CIS cohort) DFS = 42% (papillary cohort)	CR = 46% (CIS cohort) DFS = 70% (papillary cohort)	^ǂ^RFS = 58% (CIS cohort) RFS = 61% (Papillary cohort)
Efficacy outcomes at 24 mo	42%* (CIS cohort)	51% (CIS cohort)	CR = 37%* (CIS cohort) DFS = 48% (papillary cohort)	CR = 19% (CIS cohort) HG-RFS = 33% (papillary cohort)	CR = 16%* (CIS cohort) DFS = 33% (papillary cohort)	CR*=42% (CIS cohort)	^ǂ^RFS = 42% (CIS cohort) RFS = 50% (papillary cohort)
Grade III+ TRAE	0%	14% (CIS cohort)	2%	4%	13%	13%	1%

NMIBC: non-muscle-invasive bladder cancer; CIS: carcinoma *in situ*; CR: complete response; TRAE: treatment-related adverse events; Gr: grade; mo: month; IFNα2b: human interferon alpha-2b; BCG: Bacillus Calmette-Guérin; HG-RFS: high-grade recurrence-free survival; DFS: Disease-Free Survival; Q:every; IV: intravenous; N/A: not reported; NK: natural killer cells; FDA: Food and Drug Administration; μg: microgram; mg: milligram.

* This was estimated from Kaplan–Meier curves and was not clearly reported in the study results.

^ǂ^ HG-RFS was not reported in all retrospective trials, which is why it’s not included in the table.

In patients with BCG-unresponsive-papillary disease, therapeutic efficacy also varies significantly among emerging treatment modalities (Table 1). Anktiva (QUILT 3.032) demonstrated the most favorable long-term outcomes, with a disease-free survival (DFS) rate of 55% at 12 months and 48% at 24 months. Similarly, TAR-200 (SunRISe-1) showed promising durability, achieving DFS of 70% at 12 months. Adstiladrin (NTC02773849) reported high grade-RFS rates of 44% and 33% at 12 and 24 months, respectively.

Among intravesical chemotherapies, Gem/Doce has been widely used by various groups worldwide. Retrospective cohort studies have reported RFS rates of 58% at 12 months and 42% at 24 months in BCG-unresponsive CIS disease, and 61% at 12 months and 50% at 24 months in BCG-unresponsive papillary disease [15,16,17] (Table 1). The weighted mean HG-RFS (calculated from pooled trial data and adjusted for sample size) was 5-7% higher than the RFS at 12 and 24 months, although not all trials reported HG-RFS. These outcomes suggest that Gem/Doce may offer comparable or even superior efficacy to FDA-approved or investigational agents.

Several important considerations should be discussed regarding the previous efficacy comparisons, as they can help urologists make informed decisions when BSTs:

1. Cost of therapy should be considered when choosing BSTs. Based on recent cost analyses, the annual cost of therapies such as nadofaragene, Anktiva, and systemic Pembro (200 mg) is substantially higher compared to Gem/Doce, with estimates reaching $244,000, $528,300, and $204,700 respectively, versus only $3,300 for intravesical Gem/Doce [18]. This may impact access to therapy.
2. Treatment-related adverse events (TRAEs) should be considered during discussions with patients as part of a shared decision-making approach to selecting the most appropriate BSTs. Among therapies for BCG-unresponsive disease, cretostimogene monotherapy demonstrated the most favorable safety profile, with no Grade III or higher TRAE reported. In contrast, combination therapy with cretostimogene and Pembro showed the highest TRAE rate (14%), followed by TAR-200 and Pembro monotherapy (13% each). Anktiva and nadofaragene had lower TRAE rates of 2% and 4%, respectively, while intravesical Gem/Doce showed a minimal rate of 1% (Table 1). One can argue that systemic administration of Pembro in patients with localized cancer (BCG-unresponsive NMIBC) introduces the risk of immune-related systemic toxicity, thereby supporting intravesical therapy as a safer and more targeted alternative.
3. While direct head-to-head studies comparing different agents are lacking, an indirect cross-trial analysis of therapeutic agents used in BCG-unresponsive disease provides a general sense of relative efficacy and cost. These comparisons are inherently limited by differences in trial design, patient populations, dosing regimens, and outcome definitions. Nonetheless, they offer useful context for clinical decision-making.
4. Findings underscore the potential of immune-based therapies and/or combination therapies in achieving sustained disease control and also highlighting the relevance of real-world chemotherapy regimens.
5. In the absence of head-to-head comparative data, clinical decision-making should incorporate patient-specific factors such as comorbidities, renal function, allergies, and immune status. For instance, patients with impaired kidney function or systemic autoimmune conditions may be better candidates for intravesical chemotherapy rather than systemic immunotherapy. Likewise, known allergies or prior intolerance to specific agents should guide therapy selection.

In conclusion, there remains substantial room for improvement in BSTs for patients with BCG-unresponsive NMIBC. Rather than relying on indirect comparisons from phase II and III trials, head-to-head studies evaluating novel agents are urgently needed to guide clinical decision-making and optimize sequencing strategies. Equally important is the development of predictive biomarkers to identify which patients are most likely to respond to specific agents, a critical step toward personalized treatment in this challenging setting. Currently, when faced with a patient in the clinic who is either unfit for or unwilling to undergo RC, clinicians lack a clear evidence-based answer for the optimal sequencing of available therapies. While our editorial reviews most of the available options for BCG-unresponsive NMIBC, there is currently no evidence to guide sequencing after failure of another agent. Until comparative studies and biomarker-driven strategies emerge, sequencing decisions must rely on shared decision-making that balances efficacy, durability, toxicity, cost, and patient preferences.

## Data Availability

Data sharing is not applicable to this article as no new data were created or analyzed in this study.
